# Amelioration of diethylnitrosamine (DEN)-induced hepatocellular carcinogenesis in animal models *via* knockdown oxidative stress and proinflammatory markers by *Madhuca longifolia* embedded silver nanoparticles

**DOI:** 10.1039/c7ra12775h

**Published:** 2018-02-12

**Authors:** Deepika Singh, Manvendra Singh, Ekta Yadav, Neha Falls, Ujendra Komal, Deependra Singh Dangi, Vikas Kumar, Amita Verma

**Affiliations:** Department of Pharmaceutical Science, Faculty of Health Sciences, Sam Higginbottom University of Agriculture, Technology and Sciences Allahabad 211007 India; HMFA Institute of Engineering & Technology Handia Allahabad 211007 India; Department of Mechanical & Industrial Engineering, Indian Institute of Technology Roorkee Uttrakhand India; Kinapse India Scientific Services Pvt. Ltd. Gurgoan Haryana India; Natural Product Drug Discovery Laboratory, Department of Pharmaceutical Sciences, Faculty of Health Sciences, Sam Higginbottom University of Agriculture, Technology & Sciences Allahabad Uttar Pradesh 211007 India; Bio-organic & Medicinal Chemistry Research Laboratory, Department of Pharmaceutical Sciences, Faculty of Health Sciences, Sam Higginbottom University of Agriculture, Technology & Sciences Allahabad – 211007 Uttar Pradesh India amitaverma.dr@gmail.com amita.verma@shiats.edu.in

## Abstract

In hepatocellular carcinoma (HCC), primary liver cancer is primarily responsible for inflammation-related cancer as more than 90% of HCCs emerge with regard to hepatic damage and inflammation. Tenacious inflammation is known to advance and intensify liver tumours. Nanomaterials, for example, silver nanoparticles synthesized from plant-derived materials have shown great outcomes in reducing the pre-cancerous nodules and have anticancer properties. The aim of the present investigation was to biosynthesize, characterize and evaluate the anticancer activity of nanoparticles-embedded *Madhuca longifolia* extract (MLAgNPs) on an experimental model of hepatic cancer in rats. *M. longifolia* contains a high amount of flavonoids and other phenolic derivative. The silver nanoparticles synthesized by *M. longifolia* were characterized by various instruments, including UV-Vis spectrophotometry, X-ray beam diffraction, field-emission scanning electron microscopy with energy dispersive X-ray analysis, transmission electron microscopy and Fourier transform infrared spectroscopy. Liver cancer was induced to 36 Wistar rats by a single dose of diethylnitrosamine (DEN) (200 mg kg^−1^ BW). Hepatic cancer by MLAgNPs dose-dependently limited macroscopical variation compared with the DEN-induced hepatic cancer groups. The serum and liver were taken to measure the antioxidant parameters, proinflammatory cytokines and for a histopathological study. Serum hepatic and serum non-hepatic along with inflammatory cytokines were also assessed. Reduction in the levels of proinflammatory cytokines, namely tumour necrosis factor-α, interleukin-6, interleukin-1β, nuclear factor kappa beta (NF-κB), and improved membrane-bound enzyme activity were also detected. It was found that minor morphological anomalies were identified in the histopathology analysis in the MLAgNPs-treated groups. It could be concluded that silver nanoparticles introduce an extraordinary potential for use as adjuvants in hepatic cancer treatment because of their antioxidant abilities and ability to diminish inflammation in liver tissue by attenuating the NF-κB pathway.

## Abstract 2

In hepatocellular carcinoma (HCC), primary liver cancer is primarily responsible for inflammation-related cancer as more than 90% of HCCs emerge with regard to hepatic damage and inflammation.

## Graphical abstract

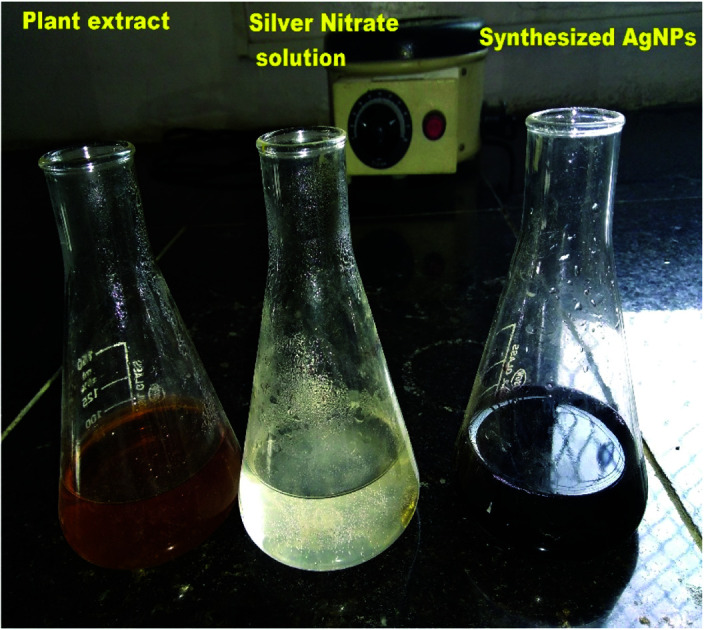

## 1. Introduction

Among the many forms of cancer, hepatocellular carcinoma (HCC) is the most well-known strong tumour and the third leading cause for disease-related mortality worldwide. The projection for hepatic cancer remains static with a 5-year survival time of <5%. It involves a multi-step process, including distinct hereditary changes causing alteration of the hepatocytes. HCC is the most widely recognized liver malignancy.^1^ Most of the instances of HCC are because of either a viral hepatitis contamination (hepatitis B or C) or cirrhosis (alcohol is the most widely recognized reason for hepatic cirrhosis). More than 600 000 individuals die from HCC every year. The frequency of this cancer is expanding and it is one of the key signs for liver transplantation.^2^ An extensive number of antitumour substances have been recently utilized for its treatment and are derived from medicinal plants. DEN is a perilous chemical which develop liver cirrhosis and is generally used to initiate hepatocarcinogenesis in rats.^3^

Novel metal nanoparticles have shown many different properties compared to other nanoparticles due to their optical, molecular and electronic properties. Metal nanomaterials have extensive optical properties due to strong electromagnetic fields. Due to these properties, metal nanoparticles are especially used in optical, imaging, sensors, cosmetics, cancer treatment, and targeted drug delivery systems. Noble metal nanomaterials are an emerging class of functional materials finding increasing acceptance in biomedical applications, such as antimicrobial agents,^4,5^ cancer therapy^6–8^ and bioimaging. Among these metals, silver and silver nanoparticles are valuable, non-reactive and not oxidized when presented to oxygen. Because of these properties, they are extraordinarily applauded and used in many biomedical applications.^9^

Silver nanoparticles provide advantages in drug targets amassing in the macrophage and due to the high binding of nanoparticles with the biological molecules, they can easily enter into mammalian cells and cause toxicities. However, synthetic-derived silver nanoparticles might have cytotoxic effects on mammalian cells, which could limit their clinical applications. Inggrid *et al.* studied the cytotoxicity of silver nanomaterials (Ag^0^-rich R NCs and Ag^+^-rich NCs) on human cells, and concluded that the Ag^0^-rich R NCs developed higher cellular toxicity by modulation of the reactive oxygen species and consequently were oxidized to Ag^+^ in the lysosomes.^10^ Zhang *et al.* proposed the photosynthetic toxicity of silver nanoclusters against green algae, namely, *Scenedesmus obliquus*, involved disrupting the electron transport chain of the light reactions and Calvin cycle of algae cells, thus unravelling the molecular mechanism of the photosynthetic toxicity of highly fluorescent silver nanoclusters to *Scenedesmus obliquus*.^11^

Different techniques are used for the preparation of silver nanoparticles; for example, physical, chemical and biological techniques. Among these techniques, utilizing a biological method is environmentally friendly, less harmful, clean and less tedious, and is considered “green science”. Nanomaterials are used for the diagnosis of cancer and its treatment. Nowadays, silver nanoparticles are utilized as a part of anticancer treatment.^12^

Biological synthesis methods cover the green synthesis of nanoparticles. These have proven to be better methods due to slower kinetics and as they offer better manipulation over control of the crystal growth and their stabilization.^13^ This method has motivated an increase in research into synthesis routes that could allow a better control of the size and shape for a wide variety of nanotechnological applications.

Advantages: biological methods for the fabrication of nanoparticles are eco-friendly and a cost effective alternative to physical and chemical methods; no requirement for energy, highly toxic chemicals or high temperature; easily transferable to large scale for the bulk synthesis of nanoparticles; offer new insights for fabricating nanoparticles using plant-derived reducing and capping agents.

Disadvantages: low production of proteins in plants, which decreases the rate of biosynthesis; when utilizing genetic engineering methods, plants cannot be manipulated for the choice of nanoparticles through optimized synthesis.^14^

*M. Longifolia* (Mahua) belongs to the family of Sapotaceae. It accomplishes a tallness of 70 feet and a fruiting age at 9–14 years and up to 60 years. It is an evergreen tree with an economic importance. Leaves of *M. longifolia* are utilized as a vegetable in India. *Madhuca longifolia* leaves are expectorant and furthermore utilized for treating chronic bronchitis, liver diseases, cancer and Cushing's disease. The stem bark is utilized to cure skin maladies, hydrocoele and skin infections. The tree is used by the tribal populations who are backwoods inhabitants and who distinctly monitor this tree. The tribes utilize the mahua tree as their source of income to make mahua drink as part of their social legacy.^15^

The extract of *Madhuca longifolia* contains different biomolecules, including alkaloids, flavonoids, phenols, tannins, glycosides, saponins, carbohydrate, proteins and enzymes, which act as reductants and are used as scaffolds during the biofabrication of silver nanoparticles in the medium. The biomolecules and enzymes present in the leaves extract possess a strong antioxidant properties and can prevent the oxidative damage to cellular apparatuses.^16^ It was already reported in the literature that some biosynthetic products of the plant play a crucial role in the reduction and stabilization of silver nanoparticles. Despite this, there is no research paper yet that has reported on the hepatic cancer activity of biosynthesized silver nanoparticles of *M. longifolia* extract.

The aim of the present investigation was to assess the defensive and restorative impacts of biogenic silver nanoparticles of *M. longifolia* leaf extract on diethylnitrosamine-induced liver cancer in animal models through contemplating the biochemical parameters and histopathology of tissues.

## 2. Materials and method

### 2.1 Chemicals

Silver nitrate and diethylnitrosamine were procured from Sigma-Aldrich (USA). The different solvents and chemicals used in the present study were of analytical grade and purchased from a local vendor.

### 2.2 Collection and authentication of the plant

The leaves of *Madhuca longifolia* were collected from Madhiyan village in the Mirzapur district, Uttar Pradesh, India. Their collection was approved and authenticated by Prof R. M. Kadam, Head of Department, Department of Botany, Mahatma Gandhi Mahavidyalaya, Latur, Maharashtra, India.

### 2.3 Preparation of the plant extract

The leaves of *Madhuca longifolia* were washed with distilled water in order to remove debris and other particulate matter. The leaves were kept in the shade for two months to dry at room temperature and then ground into fine powder by using an electrical blender. Then, 10 grams of leaf powder was boiled in 250 ml of distilled water for 2 h in Erlenmeyer flasks. The prepared extract was cooled and filtered through Whatman filter paper no. 1 by using filtration under reduced pressure. The obtained extract was placed in a refrigerator at 4 °C for further use.^17^

### 2.4 Silver nanoparticle (MLAgNPs) synthesis

**Fig. 1:**
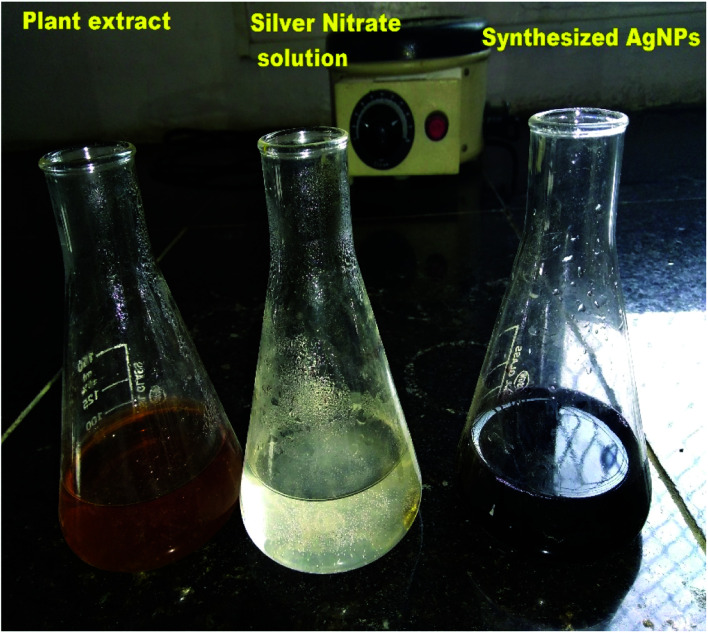
Biofabrication of silver nanoparticles by using *M. longifolia* aqueous extract.

Silver nitrate (0.017 g) was added in 100 ml of double distilled water to prepare 1 mM silver nitrate solution. Then, 10 ml of aqueous extract of *M. longifolia* was added to 90 ml of silver nitrate (1 mM) solution to obtain silver nanoparticles, as shown in Fig. 1. The prepared suspension was kept in the dark at room temperature under static conditions for nucleation of the silver nanoparticles. The suspension was subjected to centrifugation for the pellets to settle, which were then dried using a vacuum drier.^18^

### 2.5 Characterization of the silver nanoparticles

To measure the maximum production of AgNPs, UV-visible spectra were taken using a Shimadzu UV-visible spectrophotometer with a resolution of 1 nm between 200 and 800 nm. FTIR spectroscopy was used to assess the presence of different functional groups present in *M. longifolia* and the suspension was centrifuged at 8000 rpm for 20 min. The obtain pellet was ground with KBr and the spectra recorded using a Perkin Elmer FTIR spectrophotometer. An XRD instrument (Panalytical SX. Pert Pro) was used to determine the crystalline phase and purity of the MLAgNPs. The NPs were kept on a specimen stub and coated with copper sputtering to examine the surface morphology, with elemental analysis performed by FESEM (Zeiss) with EDX. The particle size and surface morphology of the MLAgNPs were measured by TEM (Hitachi) on a copper grid at an accelerated voltage of 200 kV.

### 2.6 *In vitro* cytotoxicity study of the silver nanoparticles

The *in vitro* cytotoxicity study of the silver nanoparticles included estimation by the minor modification method of Patel *et al.*^19^

### 2.7 *In vivo* hepatic cancer activity

#### 2.7.1 Animal

Swiss albino Wistar rats of both sexes, weighing between 150 and 200 g, were used for the present study. The animals were kept in the animal house of Sihas Institute of Allied and Health Science SHUATS, Allahabad at 25 °C, 45–55% humidity and with 12/12 h light/dark cycles under standard atmosphere. The animals were fed with commercially available standard pellets and water was given *ad libitum*. All the protocols for conducting the animals study were reviewed and approved according to the SHUATS University Animal Ethics Committee Guidelines and as per CSCPEA regulations with reference number IAEC/SHIATS/PA16III/SDSAV08.

#### 2.7.2 Oral toxicity study

Acute oral toxicity study was performed as per OECD Guideline-423. The rats were fasted overnight with water *ad libitum*. A dose of 5, 10 20 or 30 mg kg^−1^ BW was introduced orally to the rats. Immediately after administration of the drugs, the rats were continuously observed at least once during the first 2 h, and then again at 24 h, for any behavioural changes and in case of death. There were no deaths of Wistar rats noted till the end of the experiment, and then in those given 30 mg kg^−1^ BW of MLAgNPs. Animals did not exhibit any variation or any sign of toxic symptoms, behaviour or mortality at 30 mg kg^−1^ BW during the observation period. All the Wistar rats survived till the acute toxicity study period and no major pathological changes were found in the inner organs.

#### 2.7.3 Treatment regimen

**Fig. 2:**
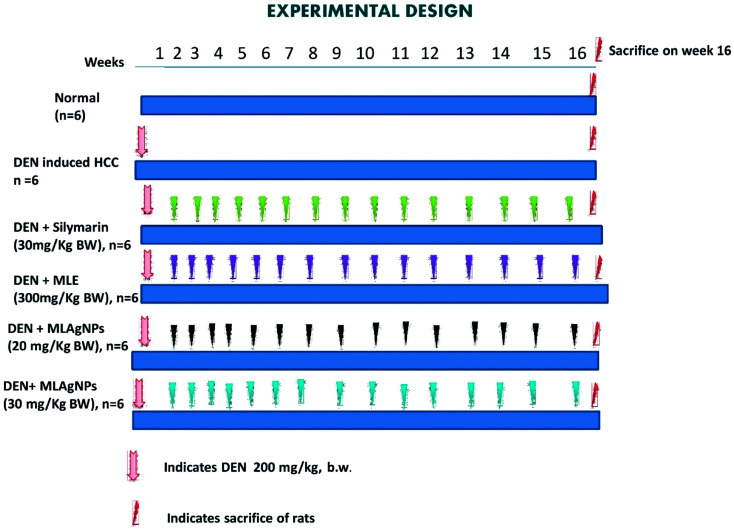
An illustration of experimental protocol.

Six groups of rats were used and each group contained six animals to study the impact of the MLAgNPs on DEN-induced hepatic cancer, as represented in Fig. 2.

Group I: received normal saline (0.9%) daily for 16 weeks.

Groups II to VIII: liver cancer was induced with a single intraperitoneal injection of DEN (200 mg kg^−1^ BW) in a saline solution.

Group II: received DEN solution only.

Group III: treated orally with MLE (300 mg kg^−1^ BW) up to a successive 16 weeks.

Group IV: treated orally with MLAgNPs (20 mg kg^−1^ BW) up to a successive 16 weeks.

Group V: treated orally with MLAgNPs (30 mg kg^−1^ BW) up to a successive 16 weeks.

Group VI: treated orally with silymarin (30 mg kg^−1^ BW) up to a successive 16 weeks.

The water, food consumption and body weight of all the animals were monitored at regular intervals. After completion of the experiment, each group of rats was sacrificed *via* cervical dislocation under an overdose of ether anaesthesia. The blood samples were collected from all the groups of rats *via* puncturing the retro-orbital sinus plexus and were kept in a labelled centrifuge tube. Serum samples were separated by centrifugation at 10 000 rpm for 15 min. Livers were excised, washed with ice-cold saline solution and stored at −90 °C. The liver samples were homogenized, and the homogenates were centrifuged in the centrifugation apparatus. The clear homogenates were collected to assess the enzymatic and non-enzymatic biochemical assays. The hepatic tissue was used for the histopathological study.^20^

#### 2.7.4 Estimation of serum biochemical parameters

Serum alanine transaminase (ALT), aspartate aminotransferase (AST), alkaline phosphatase (ALP), serum alpha fetoprotein (AFP), total proteins, total bilirubin, globulins and albumins were determined by using standard kits from Span Diagnostics, India.^21^

#### 2.7.5 Estimation of proinflammatory cytokines and inflammatory mediators

Proinflammatory cytokines and inflammatory mediators, namely TNF-α, IL-1β, IL-10 and NF-κβ, were determined using standard kits as per the manufacturers' instructions.

#### 2.7.6 Determination of lipid peroxidation

This study was done by measuring MDA in hepatic tissue, as discussed by Ohkawa *et al.*^22^

#### 2.7.7 Determination of enzymatic and non-enzymatic antioxidant enzymes

Catalase (CAT),^23^ superoxide dismutase (SOD),^24^ glutathione peroxidase (GPx),^25^ glutathione (GSH),^26^ vitamin C^27^ and glucose-6-phosphate dehydrogenase (G6PD)^28^ were determined by the reported method with minor modifications.

#### 2.7.8 Determination of membrane-bound enzymes

Na^+^/K^+^ ATPase was determined according to the method of Bonting.^29^ Ca^2+^ ATPase in the plasma membranes was measured with the procedure explained by Lotersztajn.^30^

#### 2.7.9 Histological examination

The liver tissue samples were fixed in formalin solution to preserve them and then embedded in paraffin as per standard histological procedure. Sections of six micrometres were prepared and stained with eosin and haematoxylin. The stained slides were observed under a light microscope for qualitative analysis of the hepatic histopathology.^31^

### 2.8 Statistical analysis

For estimation of the statistical analysis, GraphPad Prism 5 software was used. All the analyses were performed in triplicate. The results are expressed as the mean ± SEM and comparisons were made by one-way ANOVA with Dunnett's post-test. Differences were considered statistically significant when *p* < 0.05, *p* < 0.01 and *p* < 0.001.

## 3. Results

### 3.1 UV spectral analysis

**Fig. 3:**
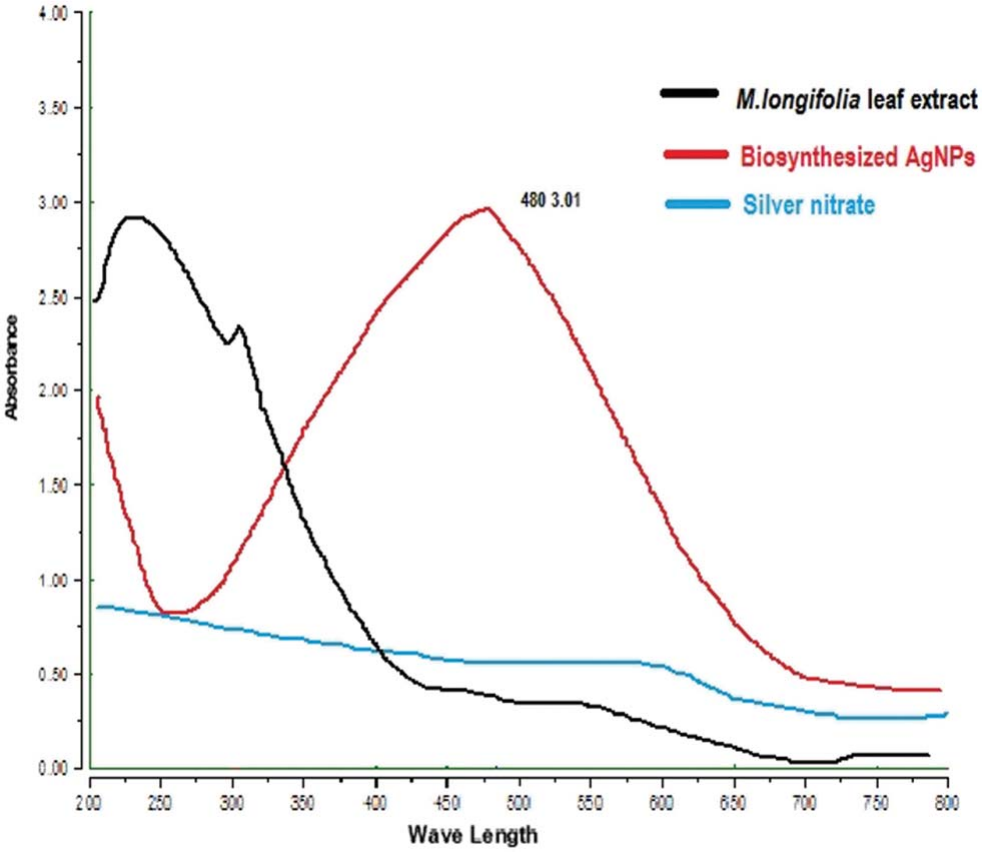
UV-Vis absorption spectra of MLAgNPs.

The formation of *M. longifolia* AgNPs was confirmed by the visual appearance of a brown colour in the suspension, as shown in Fig. 1. After 1 h of colour change, a single intense peak was obtained at 484 nm in the UV spectrophotometer due to excitation of the surface plasmon resonance (SPR), with the spectrum presented in Fig. 3. A visual colour change was obtained after the addition of plant aqueous extract to silver nitrate solution, whereupon the phytoconstituents present in the aqueous extract reduce the silver ions in AgNPs during synthesis. The higher the concentration of leaf extract, the greater the amount of bioactive compounds involved in the reduction of silver in the metal reductive process.

### 3.2 FTIR spectra analysis

**Fig. 4:**
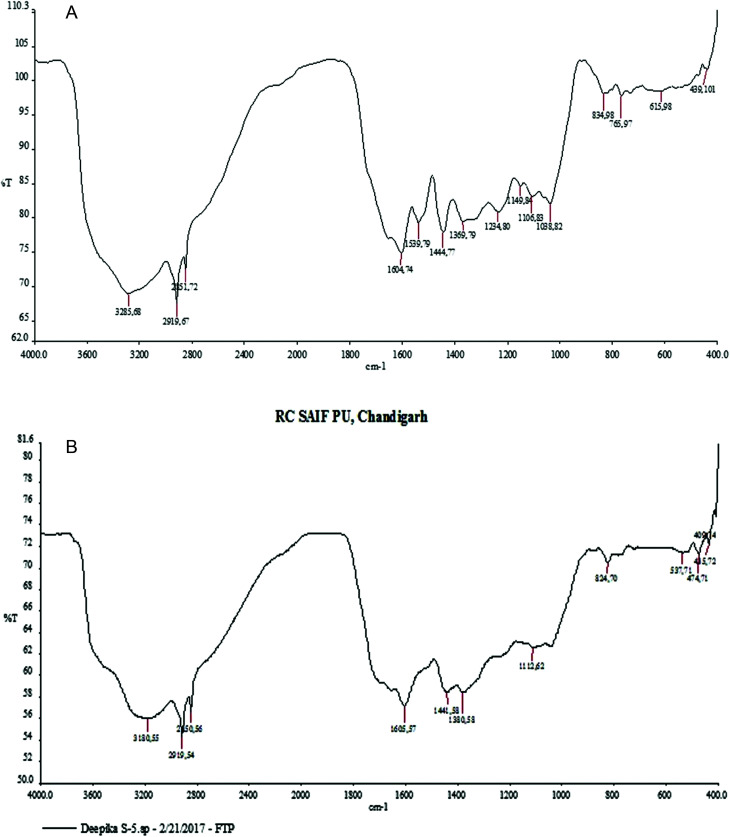
FTIR analysis: (a) MLE (b) biogenic silver nanoparticles of *M. longifolia*.

Through phytochemical screening it was observed that the aqueous leaf extract contained a carbohydrate, glycosides and flavonoid. These capping and reducing agents play a major role in the reduction of silver ions during the reaction. The presence of different functional groups and biomolecules was revealed through FTIR spectroscopy. The chemical composition of the silver nanoparticles surface was also identified by FTIR analysis. In the silver nanoparticles, intense peaks were observed at 3180 cm^−1^ assigned to phenolic O–H stretching (broad, s), while the peak at 2919 cm^−1^ was assigned to C–H (s) stretching, 1605 cm^−1^ assigned to C=C aromatic stretching, 1441 cm^−1^ assigned to aromatic C=C bending, 1112 cm^−1^ assigned to C=O stretching (s) and 824 cm^−1^ assigned to C–H bending (Fig. 4).

### 3.3 XRD pattern

**Fig. 5:**
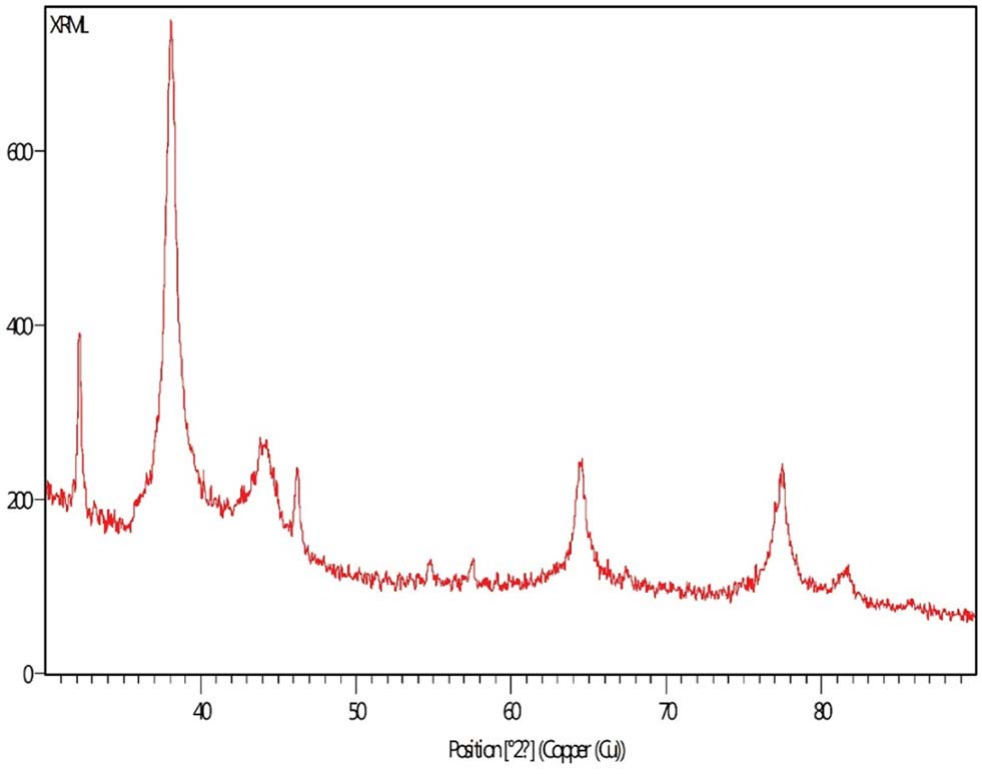
XRD spectrum of synthesized MLAgNPs.

The XRD pattern of MLAgNPs is depicted in Fig. 5. The XRD diffraction peaks at 2*θ* = 38.0339°, 44.0351°, 64.4973° and 77.5311° corresponded to the (111), (200), (220) and (311) planes as mentioned in the standard data (JCPDS file no. 01-071-4613). All the peaks were present in the figure, thus showing the crystalline structure of the silver and crystalline planes of the face-centred cubic structure.

### 3.4 FESEM

**Fig. 6:**
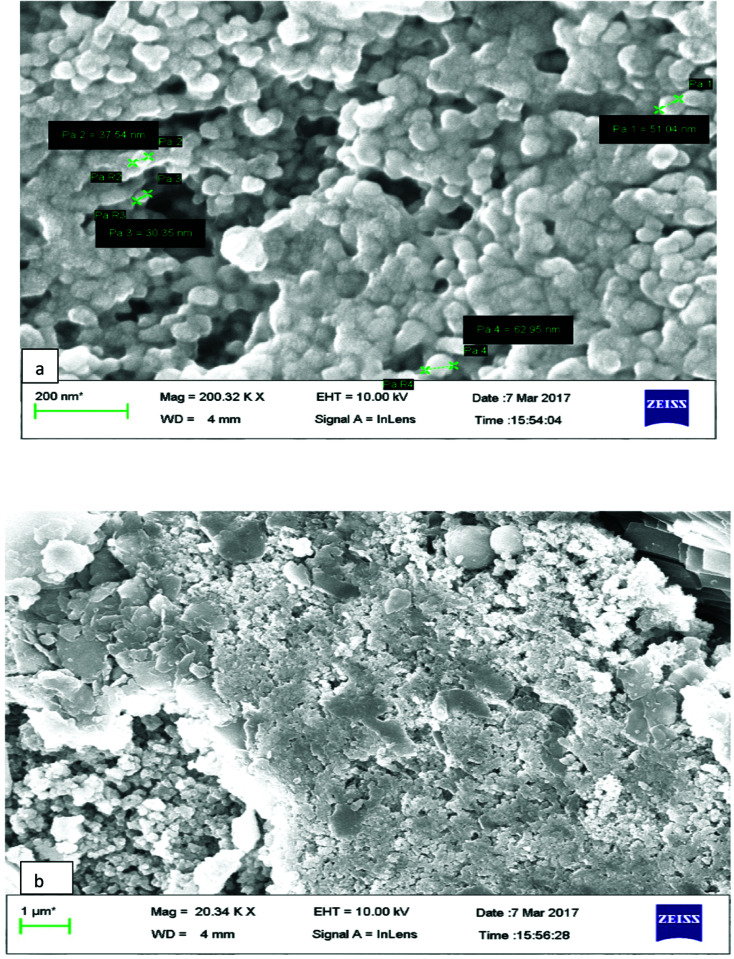
(a and b) FESEM images of synthesized MLAgNPs.

**Fig. 7:**
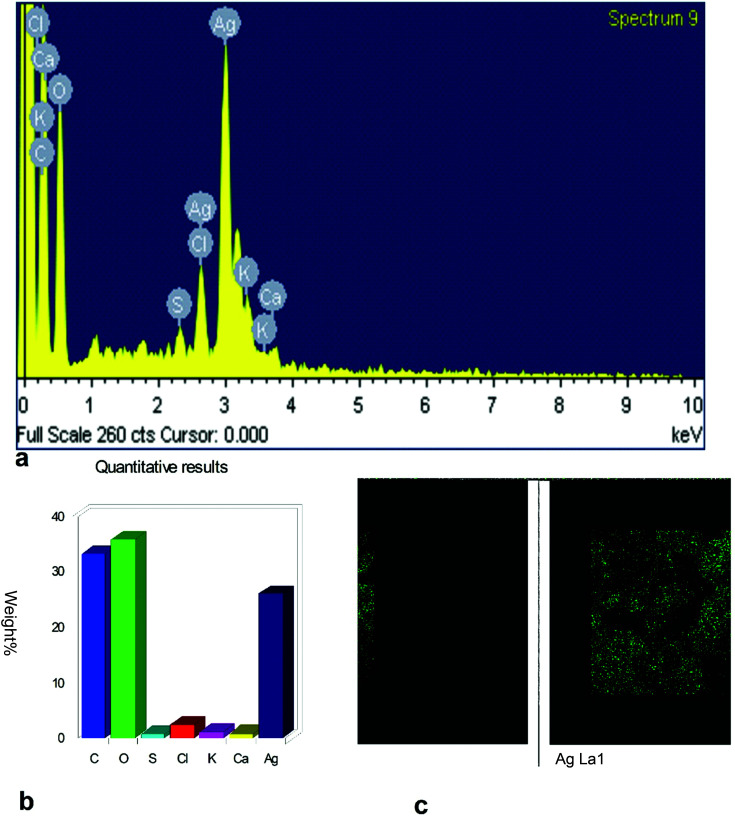
EDX spectrum of synthesized MLAgNPs.

The surface morphology of AgNPs was analyzed using an FE-SEM instrument (SUPRA-55, CARL ZEISS, GERMANY) and the images are depicted in Fig. 6. The suspension of AgNPs was placed on a clean glass plate, and the water was evaporated off to characterize the surface morphology, size and size distribution. The images showed a spherical shape of silver nanoparticles, in which the particles were enclosed by the different biocompounds and particle sizes ranging between 20 and 65 nm. The results confirmed the production of silver nanoparticles, in which the leaf aqueous extract acted as a capping and reducing agent due to the presence of biomolecules. The EDX results confirmed the presence of stoichiometry, as well as the purity and the elemental composition profile in the functionalized silver nanoparticles (Fig. 7). It revealed a strong signal for silver in the range of 2.8–3.4 keV due to the excitation of surface plasmon resonance (SPR). Other elements, such as Cl, oxygen and nitrogen, were also detected and might be due to presence of bioactive molecules present in the leaf extract of *M. longifolia*. The elemental mapping was also done by EDX to confirm the presence of silver in the silver nanoparticles. The distribution of silver on the surface of the nanoparticles confirmed the formation of AgNPs, as represented by green dots.

### 3.5 TEM

**Fig. 8:**
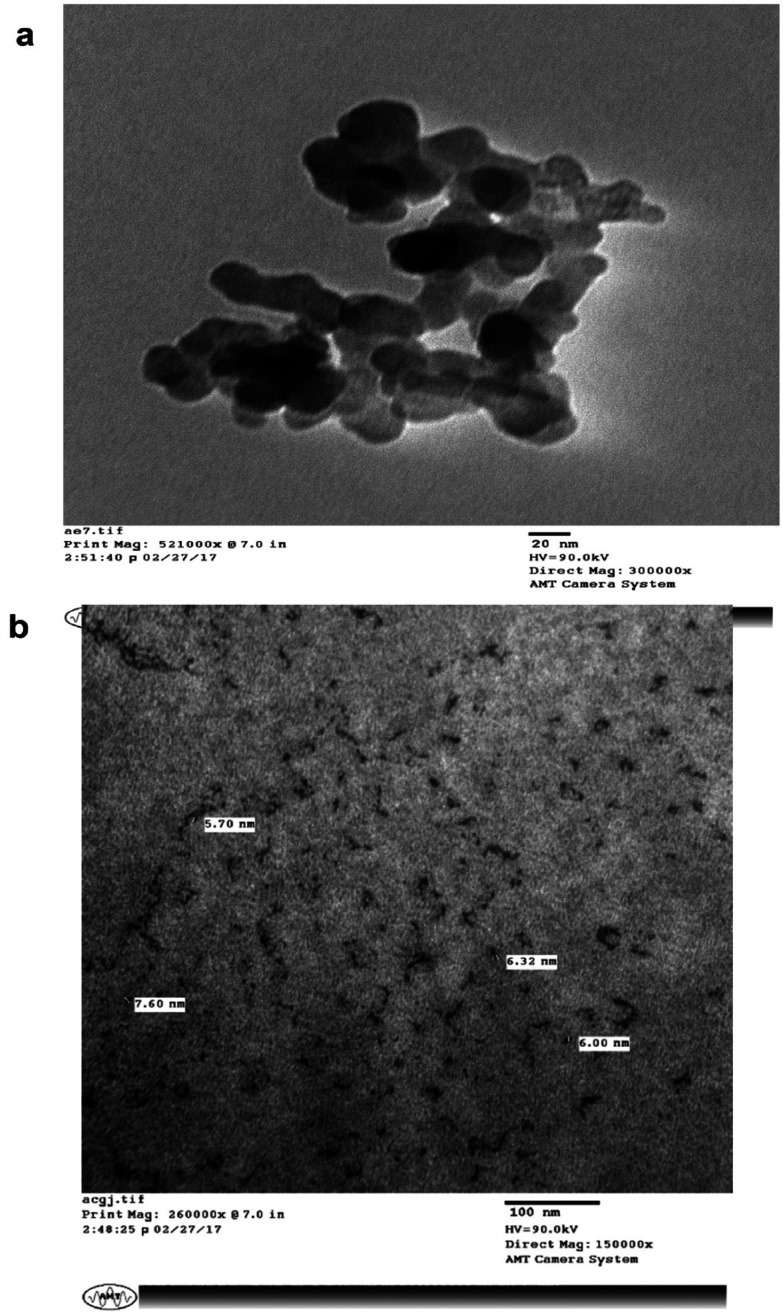
TEM images of synthesized MLAgNPs.

The surface morphology and size of the MLAgNPs was determined by TEM (Fig. 8). The TEM images showed the spherical shape of the silver nanoparticles and that they were in the size range of 5 to 20 nm, confirming the existence of silver nanoparticles. The surfaces of the nanoparticles covered with dark shades represented the secondary products, corresponding to biomolecules present in the plant leaf extract. These biomolecules were responsible for the reduction of silver salts to silver nanoparticles, and further obstruct the formation of clusters and initiate nucleation.

### 3.6 *In vitro* cytotoxic studies

**Fig. 9:**
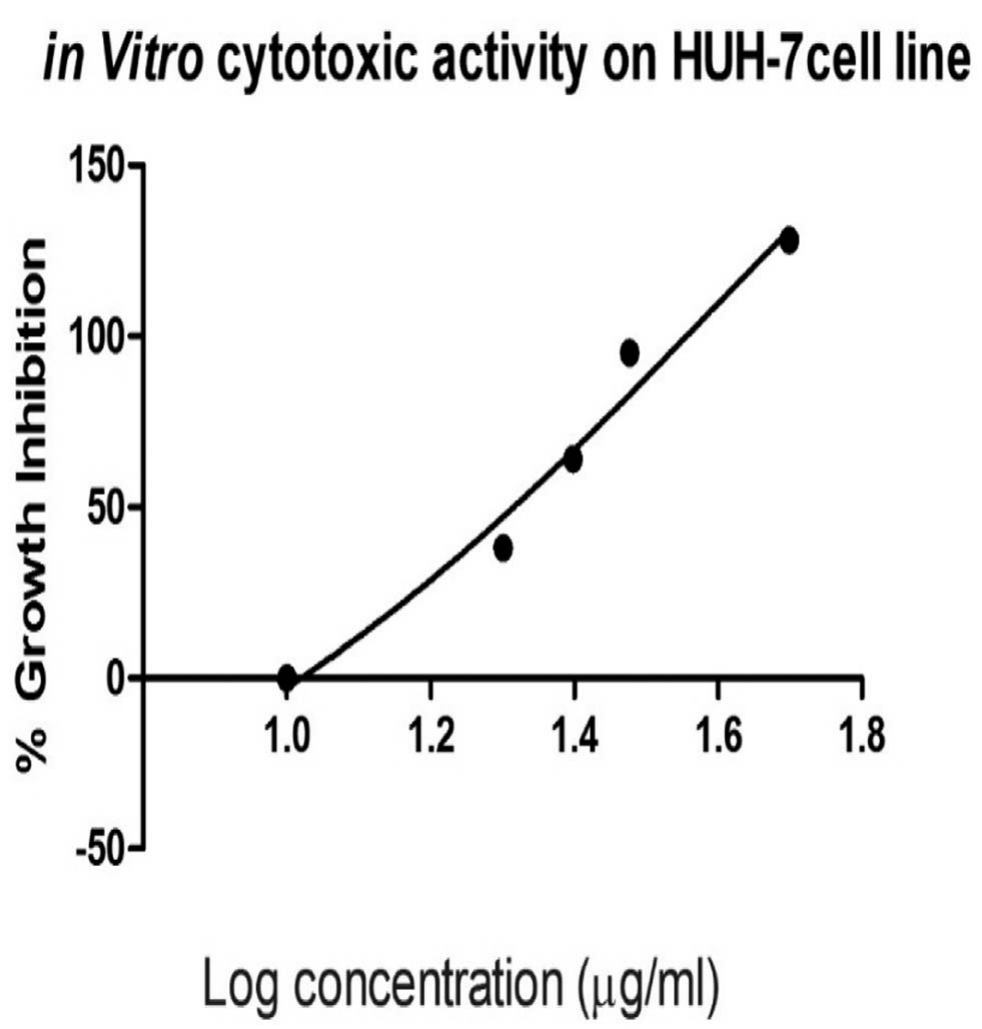
Cytotoxic activity of MLAgNPs on hepatic cancer (HuH-7 cell line).

The *in vitro* cytotoxic activity of the prepared silver nanoparticles-embedded *Madhuca longifolia* extract at different concentrations against hepatic cancer cell lines (HUH-7) was tested by MTT assay. Upon increasing the concentration of the MLAgNPs, the cell proliferation activity decreased. The IC_50_ cell inhibition value for the MLAgNPs was obtained at 41.01 μg ml^−1^. Fig. 9 presents the percentage growth inhibition of HUH-7 cell lines at different dilutions of silver nanoparticles of *Madhuca longifolia*.

### 3.7 Body weight

A significant enhancement was observed in body weight (320.62 ± 1.34) for the normal control, whereas a reduction (270.21 ± 0.34) was found in the DEN-induced group, as depicted in Table 1. The DEN-induced group when treated with MLAgNPs at a dose of 20 and 30 mg kg^−1^ BW showed upregulation (*p* < 0.001) in the weight of the bodies of the rats. A similar result was also assessed in the liver weight and mean liver weight of the DEN-induced group. The treated group with different doses of MLAgNPs showed a reduced mean liver weight in a dose-dependent manner. The DEN-induced group, when treated with silymarin, showed an improved body weight and relative liver weight when compared with the positive control group.

**Table 1:** Impact of MLAgNPs on the body weight and relative liver weight^a^

Treatment	Initial body weight	Final body weight (g)	Liver weight (g)	Relative liver weight
Control	172.54 ± 0.10	320.62 ± 1.34	8.26 ± 0.28	2.57 ± 0.28
DEN	176.62 ± 0.29	270.21 ± 0.34^z^	19.32 ± 0.45	7.15 ± 1.78^z^
DEN + sily	165.83 ± 0.08	301.46 ± 1.50^c^	9.98 ± 0.07	3.31 ± 0.61^ns^
DEN + MLE	162.35 ± 0.59	294.21 ± 1.32^c^	17.36 ± 0.78	5.90 ± 1.34^ns^
DEN + MLAgNPs20	167.90 ± 0.01	301.86 ± 0.72^c^	14.05 ± 0.13	4.65 ± 0.48^ns^
DEN + MLAgNPs30	163.45 ± 0.78	306.56 ± 1.89^c^	11.09 ± 0.01	3.61 ± 0.67^ns^

a Data are presented as the mean ± SEM (*n* = 6) and analyzed as statistically significantly (^x^*p* < 0.05, ^y^*p* < 0.01, ^z^*p* < 0.001) compared to the normal control; (^a^*p* < 0.05, ^b^*p* < 0.01, ^c^*p* < 0.001) compared to the DEN control; ns, not significant.

### 3.8 Impact of MLAgNPs on liver morphology

Table 2 depicts the total numbers of hepatic knob, average number of nodules on hepatic and the percentage of tumour incidence. An expansion of knobs on the hepatic was found in the DEN-induced groups compared to MLAgNPs, which had a lower number of knobs present on the liver in a dose-dependent manner.

**Table 2:** Effect of MLAgNPs on the development of macroscopic hepatic nodules in different groups of rats^a^

Treatment	Total no. of nodules	Tumour incidence (%)	Average number of nodules
Control	0	0	0
DEN	107	100	54.86 ± 3.01^z^
DEN + sily	14	21.38	4.16 ± 1.56^c^
DEN + MLE	83	75.32	39.18 ± 2.10^c^
DEN + MLAgNPs20	32	34.03	9.65 ± 1.54^c^
DEN + MLAgNPs30	29	31.97	8.05 ± 1.26^c^

a Normal control group did not show any visual nodule on the liver. Data are presented as the mean ± SEM (*n* = 6) and analyzed as statistically significantly (^x^*p* < 0.05, ^y^*p* < 0.01, ^z^*p* < 0.001) compared to the normal control; (^a^*p* < 0.05, ^b^*p* < 0.01, ^c^*p* < 0.001) compared to the DEN control; ns, not significant.

### 3.9 Determination of the serum marker enzyme of hepatic parameters

Table 3 illustrates the impacts of serum alanine transaminase (ALT), aspartate transaminase (AST), alkaline phosphatase (ALP) and alpha fetoprotein in the serum of the control and experimental groups of animals. Wistar rats when induced with DEN showed upregulation in all parameters with respect to the control group. MLAgNPs at different doses levels significantly (*p* < 0.001) downregulated the effects of the all serum marker enzyme compared to the toxic control group. A significance enhancement was observed in the activity of serum AFP in the DEN-induced group with respect to the normal group. Different dose strengths of MLAgNPs showed a reduction in the levels of serum AFP when treated in a dose-dependent manner. A significant reduction (*p* < 0.001) was found in the DEN + silymarin-treated group when compared with the DEN-induced group.

**Table 3:** Effect of MLAgNPs on the serum marker enzyme of the hepatic parameters^a^

Treatment	ALT	AST	ALP	AFP
Control	45.85 ± 2.41	57.23 ± 3.38	43.24 ± 2.38	132.96 ± 3.24
DEN	239.52 ± 5.17^z^	224.73 ± 6.89^z^	217.4 ± 7.73^z^	207.04 ± 5.59^z^
DEN + sily	38.93 ± 2.23^c^	69.33 ± 3.62^c^	61.26 ± 3.34^c^	146.99 ± 5.1^c^
DEN + MLE	160.23 ± 4.78^c^	178.89 ± 4.37^c^	156.07 ± 4.97^c^	187.54 ± 4.09^b^
DEN + MLAgNPs20	98.23 ± 3.78^c^	97.77 ± 3.45^c^	89.78 ± 2.28^c^	154.38 ± 3.23^c^
DEN + MLAgNPs30	68.23 ± 2.9^c^	89.56 ± 0.78^c^	76.34 ± 3.9^c^	149.89 ± 1.02^c^

a Data are presented as the mean ± SEM (*n* = 6) and analyzed as statistically significantly (^x^*p* < 0.05, ^y^*p* < 0.01, ^z^*p* < 0.001) compared to the normal control; (^a^*p* < 0.05, ^b^*p* < 0.01, ^c^*p* < 0.001) compared to the DEN control; ns, not significant.

### 3.10 Assessment of serum marker enzyme of the non-hepatic parameters

The non-hepatic enzymes parameters for all the control and experimental groups of rats are shown in Table 4. There was downregulation in the activity of all these parameters of the DEN-induced group, whereas enhancement and enzymes restoration was done by MLAgNPs in a dose-dependent manner (*p* < 0.001).

**Table 4:** Effect of MLAgNPs on the serum marker enzyme of the non-hepatic parameters^a^

Treatment	TB	TP	Albumins	Globulins	A/G ratio
Control	0.72 ± 0.09	9.78 ± 0.56	6.67 ± 0.29	3.11 ± 0.01	2.14 ± 0.03
DEN	4.02 ± 0.12^z^	5.36 ± 0.18^z^	2.78 ± 0.29^z^	2.58 ± 0.11^y^	1.08 ± 0.02^z^
DEN + sily	0.87 ± 0.11^c^	8.95 ± 0.34^c^	5.78 ± 0.67^c^	3.17 ± 0.21^b^	1.83 ± 0.07^c^
DEN + MLE	1.89 ± 0.13^c^	6.29 ± 0.09^ns^	4.22 ± 0.26^a^	2.04 ± 0.05^a^	2.03 ± 0.09^c^
DEN + MLAgNPs20	1.21 ± 0.02^c^	7.63 ± 0.5^b^	4.85 ± 0.12^c^	2.78 ± 0.04^ns^	1.87 ± 0.05^c^
DEN + MLAgNPs30	0.98 ± 0.06^c^	8.19 ± 0.54^c^	5.1 ± 0.12^c^	3.09 ± 0.16^a^	1.65 ± 0.01^c^

a Data are presented as the mean ± SEM (*n* = 6) and analyzed as statistically significantly (^x^*p* < 0.05, ^y^*p* < 0.01, ^z^*p* < 0.001) compared to the normal control; (^a^*p* < 0.05, ^b^*p* < 0.01, ^c^*p* < 0.001) compared to the DEN control; ns, not significant.

### 3.11 Effect of MLAgNPs on proinflammatory cytokines and inflammatory mediators

Table 5 demonstrates the impact of the proinflammatory cytokines on the normal control group and on the DEN-induced liver cancer group. The DEN-induced toxic group of rats had an expanded content of proinflammatory cytokines, which was lessened by the MLAgNPs treatment in a dose-dependent manner to close to the normal control rats.

**Table 5:** Effect of MLAgNPs on proinflammatory cytokines and inflammatory mediators^a^

Treatment	TNF-α	NF-κB	IL-6	IL-1β
Control	50.21 ± 2.28	159.2 ± 4.09	103.2 ± 4.29	23.26 ± 1.35
DEN	169.09 ± 4.45^z^	283.12 ± 8.98^z^	305.19 ± 8.09^z^	86.99 ± 3.98^z^
DEN + sily	60.28 ± 2.29^c^	158.78 ± 3.56^c^	106.2 ± 4.4^c^	40.02 ± 2.28^c^
DEN + MLE	100.23 ± 3.05^c^	203.89 ± 4.34^c^	200.29 ± 7.9^c^	70.12 ± 3.08^c^
DEN + MLAgNPs20	73.95 ± 2.97^c^	176.76 ± 4.78^c^	128.11 ± 3.9^c^	48.2 ± 1.55^c^
DEN + MLAgNPs30	69.29 ± 0.36^c^	170.56 ± 3.5^c^	115 ± 3.36^c^	45.29 ± 1.3^c^

a Data are presented as the mean ± SEM (*n* = 6) and analyzed as statistically significantly (^x^*p* < 0.05, ^y^*p* < 0.01, ^z^*p* < 0.001) compared to the normal control; (^a^*p* < 0.05, ^b^*p* < 0.01, ^c^*p* < 0.001) compared to the DEN control; ns, not significant.

### 3.12 Evaluation of lipid peroxidation activity

A significant enhancement was observed in MDA content (a biomarker of LPO) during DEN-induced hepatic cancer in Wistar rats. The administration of MLAgNPs at different doses significantly (*p* < 0.001) downregulated the LPO level in a dose-dependent manner (Table 6). When LPO level was determined, DEN + sily demonstrated a downwards direction of the MDA level with respect to the normal group.

**Table 6:** Effect of MLAgNPs on lipid peroxidation activity^a^

Treatment	MDA
Control	22.01 ± 1.02
DEN	42.18 ± 0.15^z^
DEN + sily	20.11 ± 0.07^c^
DEN + MLE	31.78 ± 1.08^c^
DEN + MLAgNPs20	22.89 ± 1.02^c^
DEN + MLAgNPs30	20.22 ± 1.67^c^

a Data are presented as the mean ± SEM (*n* = 6) and analyzed as statistically significantly (^x^*p* < 0.05, ^y^*p* < 0.01, ^z^*p* < 0.001) compared to the normal control; (^a^*p* < 0.05, ^b^*p* < 0.01, ^c^*p* < 0.001) compared to the DEN control; ns, not significant.

### 3.13 Effect of silver nanoparticles on enzymatic and non-enzymatic antioxidants

The levels of SOD, catalase, GPx, GSH, G6PD and vitamin C were significantly decreased (*p* < 0.001) in the DEN-induced toxic group with respect to the control group of rats (Table 7). After administration of MLAgNPs in a dose-graded response manner, a significant (*p* < 0.001) increment in the levels of all the enzymatic and non-enzymatic antioxidants was noted. A better response was also seen in the enzymes after the administration of the standard drug in the DEN-induced group.

**Table 7:** Effect of MLAgNPs on enzymatic and non-enzymatic antioxidant profiles^a^

Treatment	Catalase	SOD	GPx	GSH	G6PD	Vitamin C
Control	93.99 ± 3.36	7.38 ± 0.18	11.56 ± 0.56	50.29 ± 0.37	9.89 ± 0.22	4.28 ± 0.06
DEN	50.28 ± 2.36^z^	2.63 ± 0.09^z^	4.9 ± 0.28^z^	15.38 ± 0.37^z^	2.56 ± 0.14^z^	40.67 ± 0.75^z^
DEN + sily	74.28 ± 3.78^c^	7.56 ± 0.15^c^	10.06 ± 0.09^c^	45.38 ± 0.57^c^	8.06 ± 0.1^c^	22.11 ± 0.11^c^
DEN + MLE	60.11 ± 2.9^ns^	4.78 ± 0.12^c^	7.04 ± 0.65^b^	31.66 ± 0.78^c^	5.37 ± 0.34^c^	28.2 ± 1.28^c^
DEN + MLAgNPs20	67.12 ± 3.09^b^	6.03 ± 0.27^c^	8.56 ± 0.23^c^	37.2 ± 0.79^c^	6.69 ± 0.07^c^	21.81 ± 1.03^c^
DEN + MLAgNPs30	69.36 ± 1^c^	6.24 ± 0.15^c^	9.05 ± 0.58^c^	40.12 ± 0.02^c^	7.13 ± 0.67^c^	20.98 ± 0.36^c^

a Data are presented as the mean ± SEM (*n* = 6) and analyzed as statistically significantly (^x^*p* < 0.05, ^y^*p* < 0.01, ^z^*p* < 0.001) compared to the normal control; (^a^*p* < 0.05, ^b^*p* < 0.01, ^c^*p* < 0.001) compared to the DEN control; ns, not significant.

### 3.14 Membrane-bound activities

Significant reductions were seen in the level of Na^+^/K^+^ ATPase and Ca^2+^ ATPase in the DEN-induced liver cancer group rats when compared with the normal control Wistar rats. The administration of MLAgNPs in a dose-graded response manner showed an enhancement in Ca^2+^ ATPase levels. A practically comparative result of lessening was observed for the Na^+^/K^+^ ATPase activity in the liver tissue of the DEN-induced group rats, as shown in Table 8. Silymarin (standard drug), when introduced to the DEN-induced group, showed a significantly raised level of ATPase and restored the content in rats.

**Table 8:** Effect of MLAgNPs on the membrane-bound activities^a^

Treatment	Na^+^/K^+^ ATPase	Ca^2+^ ATPase
Control	4.42 ± 0.12	9.93 ± 0.28
DEN	2.57 ± 0.18^z^	4.29 ± 1.02^z^
DEN + sily	4.17 ± 0.05^c^	8.01 ± 0.09^c^
DEN + MLE	3.45 ± 0.23^a^	5.66 ± 0.21^ns^
DEN + MLAgNPs20	3.97 ± 0.03^c^	7.23 ± 0.59^b^
DEN + MLAgNPs30	4.02 ± 0.2^c^	7.76 ± 0.28^c^

a Data are presented as the mean ± SEM (*n* = 6) and analyzed as statistically significantly (^x^*p* < 0.05, ^y^*p* < 0.01, ^z^*p* < 0.001) compared to the normal control; (^a^*p* < 0.05, ^b^*p* < 0.01, ^c^*p* < 0.001) compared to the DEN control; ns, not significant.

### 3.15 Histology of liver tissue

**Fig. 10:**
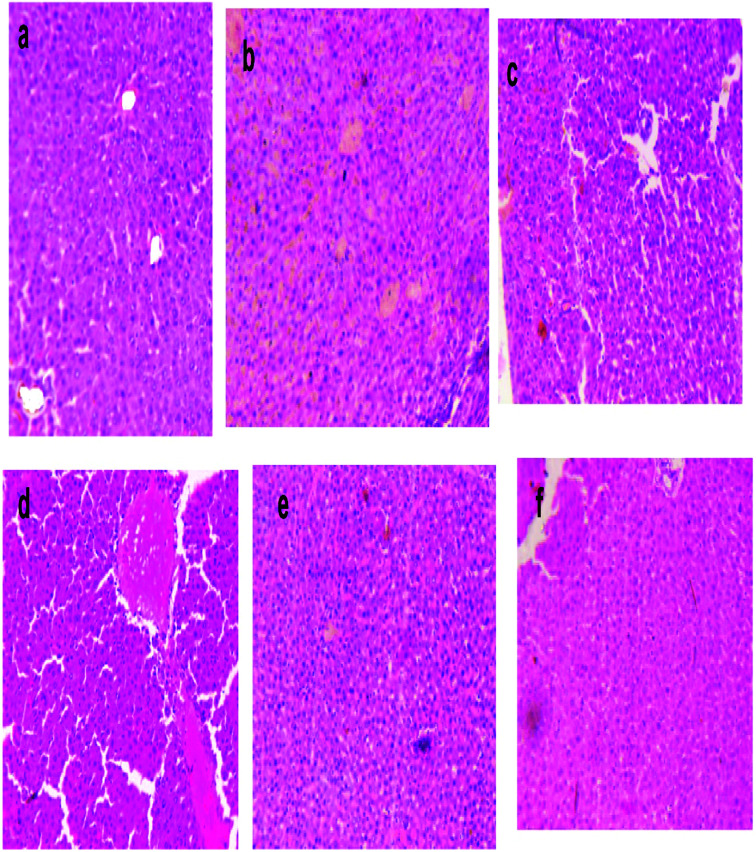
Photomicrograph of eosin–haematoxylin stained histological liver section area for (a) normal control group, (b) DEN induced liver cancer group (c) DEN + silymarin (d) DEN + MLE (e) DEN + MLAgNPs (20 mg kg^−1^ BW), and (f) DEN + MLAgNPs (30 mg kg^−1^ BW).

The liver sections of the rats were stained with eosin and haematoxylin, as illustrated in Fig. 10. The liver of normal control rats showed a well-defined structure of the liver, in which mono-nucleated cells have a regular border and are in contact with surrounding cells. The DEN-induced liver cancer group revealed an irregular cellular structure with divided nuclei, which had lost their spherical shapes, while a focal necrosis, hepatocellular adenoma and hyperplastic knob were present on them.^32^ When this group was exposed or treated with silymarin it induced huge changes in the section of liver, which then only showed the dilation of blood sinusoids.^33^ DEN-induced rats, when treated with different dose levels of MLAgNPs, seemed to invert the auxiliary changes observed in the liver, with neoplastically transformed cells and showed a certain level of improvement when compared to the DEN-induced rats.

## 4. Discussion

Carcinogenesis is a general term meaning the advancement of neoplasia. DEN is an outstanding hepatocarcinogen typically used to initiate liver malignancy in living models. It has been demonstrated that upon essential metabolic enactment, DEN delivers the promutagenic adducts *O*-6-ethyl deoxyguanosine and *O*-4 and *O*-6-ethyl deoxythymidine, which generate DNA chain harm, depurination or attached to DNA and regularly produce a miscoding in DNA, and leads to the initiation of liver carcinogenesis taking place.^34^ A rat model was used to examine diethylnitrosamine-initiated carcinogenesis in rats built up in our research facility. The neurotic changes within the movement of tumours and their hindrance are relied upon to be reflected in the natural and histological parameters of the host framework.^35^

Silver nanoparticles were prepared by aqueous leaves extract of *M. longifolia*. The development and stability of the prepared silver nanoparticles in the colloidal solution were characterized by UV-Vis spectrophotometry, FTIR, X-beam diffractometry (XRD), FESEM with EDX and TEM.^36^

Silver nanoparticles play a noteworthy part in the field of nanotechnology and nanomedicine. The AgNPs prepared in this procedure were found to be effective anticancer agents against DEN-induced hepatic cancer animal models.

One of the manifestations related with hepatocellular carcinoma is weight reduction and tissue destruction. There was a sharp abatement in the body weight and an expansion in the liver weights at 16 weeks in animals. This is despite the fact that it was normal that treatment with MLE, MLAgNPs and silymarin could alter the trend.^37^

An *in vitro* cytotoxic study confirmed that the silver nanoparticles had an effect on the hepatic cancer cell line in a dose-dependent way. This might be due to the interaction of the nanoparticles with the proteins of the cell to produce some alterations due to its low stability and due to the highly reactive nature of silver ions.^38^

A significant enhancement was observed in the levels of all the serum markers enzymes of the DEN-induced group when compared to those of the normal group. The MLAgNPs-treated group restored all the values of serum enzymes back to normal levels. These serum markers enzymes are used to detect the hepatic damage with HCC injuries, where all these enzymes are discharged from the injured hepatocytes into the circulatory system, since these are essentially restricted in the liver. Serum bilirubin is an intracellular enzyme available in the liver and is an indicator for hepatic damage. MLAgNPs showed they could contribute to the stability of the membrane or repair liver damage by maintaining the functional integrity of the membrane in the liver. They reduce the spillage of marker enzymes through the layers of membrane, giving hepatoprotective activity and hindering carcinogenesis.^39^

In the DEN-induced group, defective protein synthesis took place, which caused hepatic cell damage in turn, HCC. Silver nanoparticles subsequently stopped the deleterious effect of these non-hepatic enzymes and initiated the synthesis of protein. AFP is a typical gold marker diagnosed in the serum of HCC patients and germ cell malignancies.^40^ Elevated levels of AFP were seen in the Wistar rats when presented to hepatocarcinogen, which is a glycoprotein whose molecular weight is 70 kDa, situated at chromosome 4q11-q13, showing allelic losses in liver cancer. There are a few reports that demonstrate that AFP assumes a fundamental role in the control of tumour development, cell separation and in mediating multiplication of human hepatoma cells, conceivably through AFP receptors. Elevated levels of AFP were seen in the DEN-induced group, demonstrating the existence of hepatic cell carcinoma.^41^ The plausible clarifications for the expansion of AFP synthesis by neoplastic hepatocytes are either an expanded interpretation of AFP gene or post-translational change influencing AFP formation. MLAgNPs fundamentally lessened the AFP level compared with the DEN-initiated liver cancer group.

A few studies have demonstrated that liver is the main centre for the production of proinflammatory cytokines (IL-1β, TNF-α and IL-6) through blood (lymphocytes and monocytes) along with Kuffer cells, human and murine hepatocytes. Kuffer cells assume a vital part in the arrival of IL-6 through modification of MyD88-subordinate NF-κB triggering, which additionally stimulates IL-1α from hepatocytes. DEN demonstrates an impact on the Kuffer cells and also mediates the NF-κB triggering MyD88-subordinate; which initiates the discharge of IL-6 and induces hepatic cancer.^42^ Our investigation affirmed the restraint of IL-6 and furthermore the decreased secretion of IL-6. Another proinflammatory cytokine, TNF-α which is considered as an NF-κB activator, produced during the inflammatory response, assumes a noteworthy part in inflammation and is termed as a cell multiplication accelerator.^43^ Our results demonstrated the lower content of TNF-α in DEN-induced hepatic cancer Wistar rats, which was downregulated by MLAgNPs, affirming the improved level of TNF-α close to the level in the control group rats. Advanced investigation for the affirmation of the mode of action need broad research to be undertaken by the researcher to build up knowledge of the mechanism.

Lipid peroxidation is regarded as the traditional marker of oxidative stress. A few occurrences affirmed a conceivable system for destruction of the corpuscle in LPO, which is generated by free radicals. Free radicals assimilate the electron from the plasma membrane and begin harming the cells with lipid destruction. The steady production of oxygen responds with the unsteady unsaturated fat, producing a peroxy unsaturated fatty radical, which keeps on responding with another free radical and generates an eccentric chain of lipid peroxide during the oxidative stress. During DEN-initiated hepatic cancer, MDA responds to different free radicals and starts the oxidative stress, which is responsible for carcinogenesis.^44^ The upgraded level of LPO after DEN administration enhanced the production of free radicals, which was affirmed by the decreased level of endogenous antioxidants. The enhanced level of LPO concentration was deduced by the MLAgNPs dose dependently and ascribed to their free radical scavenging impact.^45^

Reactive oxygen species and oxidative stress are the main factor responsible for the hepatic tissue damage, which leads to developing a tumour, which in turn causes HCC. The reason behind the free radical is GSH and its metabolizing enzymes.^46^ The antioxidant enzymes are the first-line defence mechanism against such damage and thus provide protection against the deteriorating outcome. The enzymatic and non-enzymatic antioxidants researched to date are known to diminish the oxidative stress by decreasing the production and assembling of superoxide radicals (O_2_). Protection was seen by the *M. longifolia*-embedded silver nanoparticles in a dose-dependent manner and then reverted back to the DEN-induced deduction in all the antioxidants.^47^ The protective effect was shown by the flavonoids present in the silver nanoparticles on the cell, which modulated the gene expression, and all enzymes level activities, which were directly involved in antioxidant defence and the glutathione effect.^48^ It might be possible that the different phytoconstituents of the plant are associated with the free scavenging radicals from the tissues, consequently, decreasing oxidative stress.^49^

A few exploratory studies have affirmed that membrane-bound agents, such as Na^+^/K^+^ ATPase and Ca^2+^ ATPase, transfer ions over the cell membrane by utilizing the energy in the form of ATP. The production of lipid peroxidation changes the basic structure of the cell membrane, which further specifically influences the membrane-bound ATPase action.^50^ DEN-induced liver cancer rats affirmed the lessening of the membrane-bound enzyme and MLAgNPs treatment raised the Na^+^/K^+^ ATPase and Ca^2+^ ATPase by means of balancing out the intrusion in the potassium and calcium digestion.^51^ So, we officially affirmed that the MLAgNPs administration maintains the concentration of lipid peroxidation in DEN-induced HCC groups, protects the hepatocytes from damage and is responsible for cell membrane integrity.^52^

## 5. Conclusion

Our outcomes have demonstrated that the bioengineered silver nanoparticles of *M. longifolia* leaves extract cause *in vitro* and *in vivo* apoptosis of hepatic cancer through an ROS pathway and are promising agents in liver carcinogenesis.

## Conflicts of interest

The authors have none potential conflict of interests.

## Abbreviation

AgNPs: Silver nanoparticles
*M. longifolia*
: *Madhuca longifolia*
DEN: Diethylnitrosamine
MLAgNPs: *Madhuca longifolia*-embedded silver nanoparticles
MLE: *Madhuca longifolia* extract
FESEM: Field-emission scanning electron microscopy
EDX: Energy dispersive X-ray analysis
FTIR: Fourier transform infrared spectroscopy
SPR: Surface plasmon resonance
TEM: Transmission electron microscopy
ALT: Alanine amino transferase
AST: Aspartate amino transferase
ALP: Alkaline phosphatise
GPx: Glutathione peroxidase
SOD: Superoxide dismutase
HCC: Hepatocellular carcinoma
AgNO_3_: Silver nitrate
DMSO: Dimethyl sulfoxide
MTT: (3-(4,5-Dimethylthiazol-2-yl)-2,5-diphenyltetrazolium bromide)
AFP: Alpha feto protein
G6PD: Glucose-6-phosphate dehydrogenase
MDA: Malondialdehyde
NADP: Nicotinamide adenine dinucleotide phosphate
NADPH: Nicotinamide adenine dinucleotide phosphate-oxidase
LPO: Lipid peroxidation

## Supplementary Material
